# Targeted genome modifications in soybean with CRISPR/Cas9

**DOI:** 10.1186/s12896-015-0131-2

**Published:** 2015-03-12

**Authors:** Thomas B Jacobs, Peter R LaFayette, Robert J Schmitz, Wayne A Parrott

**Affiliations:** Institute for Plant Breeding, Genetics and Genomics, University of Georgia, Athens, Georgia 30602 USA; Department of Crop and Soil Sciences, University of Georgia, Athens, GA 30602 USA; Center for Applied Genetic Technologies, University of Georgia, Athens, GA 30602 USA; Department of Genetics, University of Georgia, Athens, GA 30602 USA; Present address: Boyce Thompson Institute for Plant Research, Ithaca, NY 14853 USA

**Keywords:** CRISPR/Cas9, Plant transformation, Soybean, Genomic engineering, Gene targeting, Hairy roots

## Abstract

**Background:**

The ability to selectively alter genomic DNA sequences *in vivo* is a powerful tool for basic and applied research. The CRISPR/Cas9 system precisely mutates DNA sequences in a number of organisms. Here, the CRISPR/Cas9 system is shown to be effective in soybean by knocking-out a green fluorescent protein (GFP) transgene and modifying nine endogenous loci.

**Results:**

Targeted DNA mutations were detected in 95% of 88 hairy-root transgenic events analyzed. Bi-allelic mutations were detected in events transformed with eight of the nine targeting vectors. Small deletions were the most common type of mutation produced, although SNPs and short insertions were also observed. Homoeologous genes were successfully targeted singly and together, demonstrating that CRISPR/Cas9 can both selectively, and generally, target members of gene families. Somatic embryo cultures were also modified to enable the production of plants with heritable mutations, with the frequency of DNA modifications increasing with culture time. A novel cloning strategy and vector system based on In-Fusion® cloning was developed to simplify the production of CRISPR/Cas9 targeting vectors, which should be applicable for targeting any gene in any organism.

**Conclusions:**

The CRISPR/Cas9 is a simple, efficient, and highly specific genome editing tool in soybean. Although some vectors are more efficient than others, it is possible to edit duplicated genes relatively easily. The vectors and methods developed here will be useful for the application of CRISPR/Cas9 to soybean and other plant species.

**Electronic supplementary material:**

The online version of this article (doi:10.1186/s12896-015-0131-2) contains supplementary material, which is available to authorized users.

## Background

Methods to specifically target and modify DNA sequences are indispensable for basic and applied research. Recently, the type II bacterial clustered, regularly interspaced, short palindromic repeats (CRISPR) system emerged as a simple and efficient tool to target and modify DNA sequences of interest in a variety of organisms, including; cultured human cells [1,2], zebrafish embryos [3], yeast [4], mice [5], and plants such as rice [6-9], *Arabidopsis thaliana* [10], maize [11] and liverwort [12].

There are two components to the CRISPR system: a nuclear-localized CRISPR-associated (Cas) 9 protein and a guide RNA (gRNA). Cas9 is a large protein containing two nuclease domains, and the most commonly used one is derived from *Streptococcus pyogenes*. The gRNA is a synthetic 100 nucleotide (nt) RNA molecule, of which the first approximately 20 nt are the targeting site, and the 3′ end forms a hairpin structure that interacts with the Cas9 protein [13]. Cas9 and the gRNA interact to identify DNA sequences complementary to the gRNA and generate a DNA double-strand break (DSB).

When a DNA DSB occurs in eukaryotic cells, the imprecise repair mechanism, non-homologous end joining (NHEJ), can result in the insertion and/or deletion of sequences at the breakage site, typically resulting in frame-shift mutations [14]. In plants, such targeted DSBs can be used to knock-out genes [15,16], modify gene expression by disrupting promoter sequences [17], or insert transgenes at a specific location via homologous recombination [18-22].

This work characterizes and further extends the use of CRISPRs for the genetic modification of soybean genes. CRISPR vectors targeting 11 loci were introduced into soybean via *Agrobacterium rhizogenes* to generate transgenic hairy roots. Custom-amplicon sequencing of DNA from these roots show that genetic modifications were made in 95% of the tested events. Modifications were also detected in somatic embryo cultures, and these should result in soybean lines with germinal modifications. Differences between *Agrobacterium*- and particle bombardment-mediated transformation were observed and may be important considerations for transformation experiments. To facilitate CRISPR mutagenesis efforts, a series of CRISPR vectors and a novel gRNA cloning method were produced.

## Results and discussion

### Knock-out of a GFP transgene

**Figure 1:**
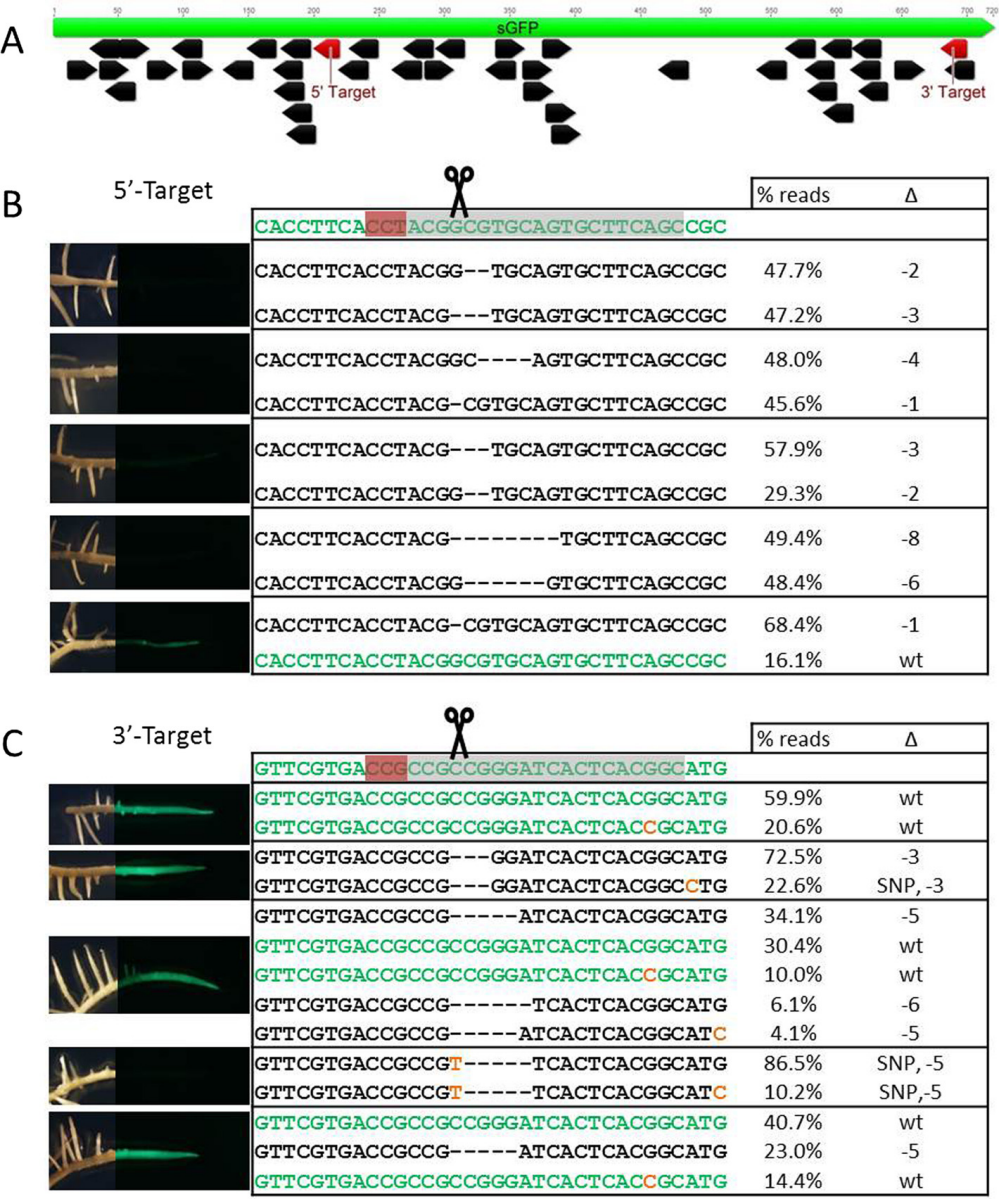
**Cas9 targeting of a GFP gene in soybean hairy roots. (A)** Schematic showing the targeted GFP sequences. The targets were designed to the negative strand of GFP. Black arrows are all possible GN_20_GG target motifs. GFP imaging and amplicon sequencing of representative **(B)** C9 + GFP 5′ target events and **(C)** C9 + GFP 3′ target events. Each panel is an independent event and blue-light images were overlaid onto white-light images of roots. The same magnification was used for all images. Wild-type sequences are in green, deletions are shown as dashes, and SNPs are shown in orange. The targeted sequences are highlighted in grey and the PAM is highlighted in red. Percentages next to sequences indicate the number of reads with sequence over the number of total reads sequenced. On average, there were 4,282 and 8,409 reads per event from the 5′-target and 3′-target events, respectively.

The first test of the CRISPR system in soybean was with a GFP (*Green Fluorescent Protein*)-expressing soybean line, as GFP knock-outs are easily observed by a loss of fluorescence. Two GFP-targeting gRNA vectors were designed; one gRNA was designed to target the 5′ end of *GFP* (5′-target) and a second was designed to target the 3′ end (3′-target) (Figure 1A). The vectors were introduced into the GFP line via *A. rhizogenes* to produce hairy roots. Fifteen out of 17 5′-target events and four of the 22 3′-target events were knock-outs as evident by a loss of fluorescence under blue-light (Additional file 1). Controls containing either Cas9 or the gRNAs alone, all fluoresced (Additional file 1). Since the GFP soybean line used is homozygous for GFP, these results show that the CRISPR system is able modify both GFP alleles, which is the only way to get loss of fluorescence.

Custom-amplicon sequencing was used to determine the genetic modifications at the *GFP* transgene. The most abundant mutations at the 5′-target were short (1-21-nt) deletions (Figure 1, Additional file 2). For event 10, a wild-type sequence was observed in 16% of the reads, which is consistent with fluorescent imaging (Figure 1 and Additional file 1). The 3′-target is less efficient; wild-type sequences were observed in seven of the events, with one event being completely unmodified (Additional file 2). Events with wild-type and modified sequences may be due to a single GFP allele being modified, or to the presence of chimeric tissues. Four of the 3′-target events contained SNPs and one event contained a T insertion, whereas the 5′-target events did not contain any SNPs or insertions. A single SNP at the 3′-target was routinely observed in the modified events and Cas9 control and may be due to errors during library preparation or sequencing.

### Modifying a soybean gene

Given the successful modifications of the GFP targets, the next attempt was to modify the single-copy soybean gene, Glyma07g14530, which is a putative glucosyl-transferase. Glyma07g14530 custom amplicons from ten independent events were sequenced, and these showed a variety of mutations, including deletions, SNPs, insertions, and replacements (Additional file 2). Replacements are defined as two or more bases that were incorporated after a deletion event. Three events contained only modified sequences, six events had both wild-type and modified sequences, and one event had no modifications. These results indicate that both mono- and biallelic modifications were made and/or chimeric tissues were present.

### Targeting gene pairs

**Figure 2:**
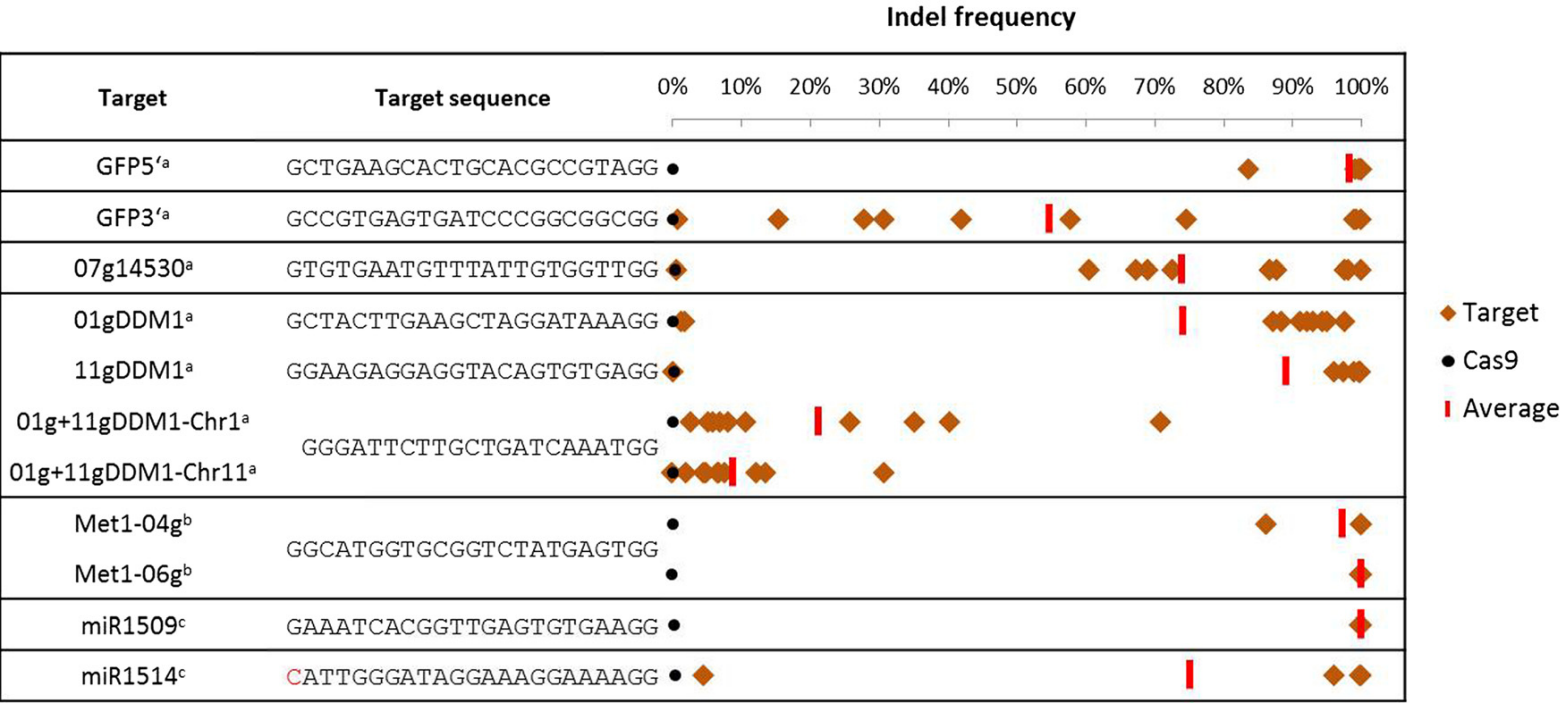
**Modification efficiency for hairy root events.** Custom-amplicon sequencing was used to measure indel frequency for each of the targeting constructs. Individual events are in orange triangles, the Cas9-tranformed control is in black circles, and average indel frequencies are vertical red bars. The miR1514 target sequence has a single mismatch to the gRNA in red. ^a^ n = 10, ^b^ n = 5, ^c^ n = 4.

**Figure 3:**
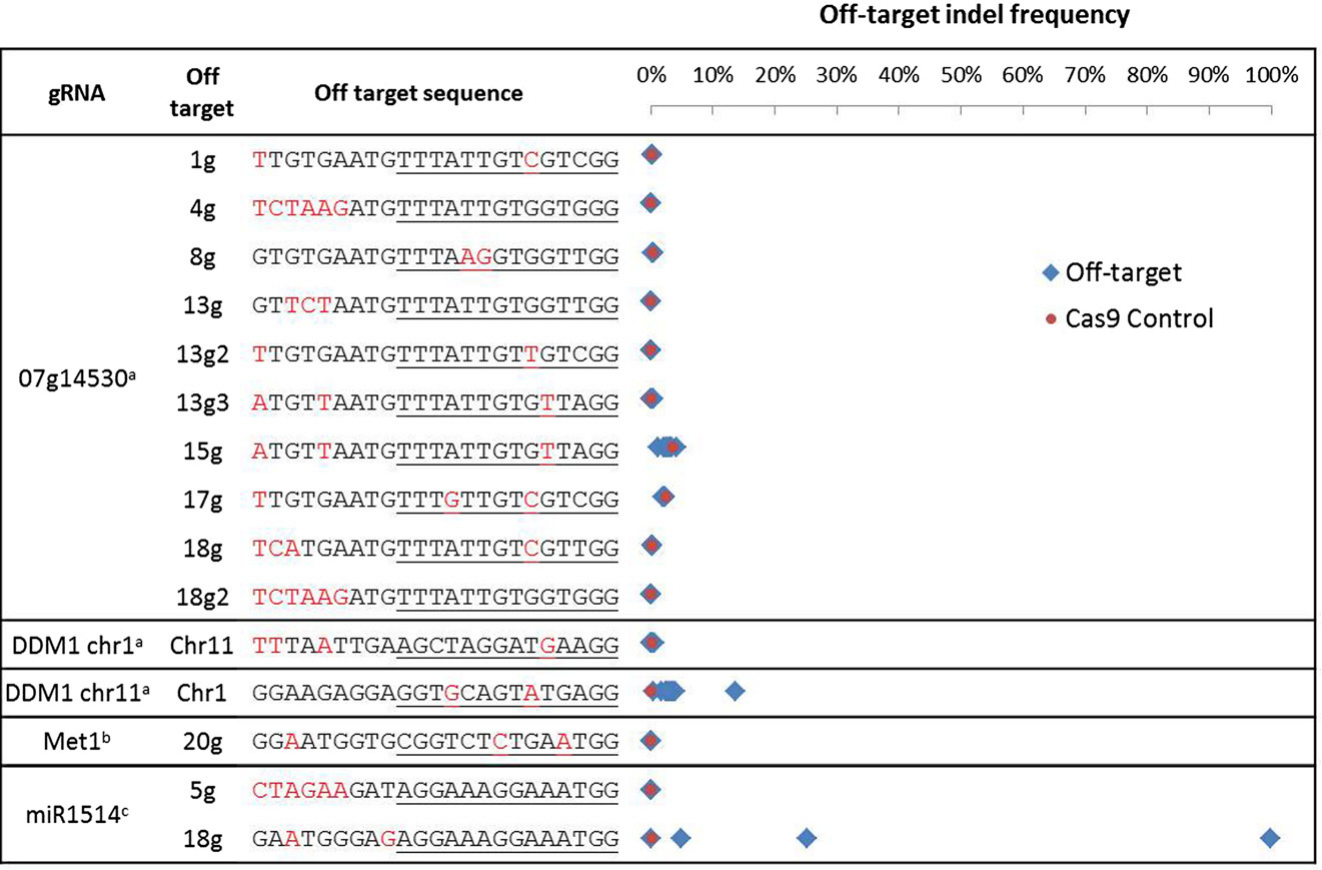
**Off-target indel frequency for hairy-root events.** The measured indel frequency is represented by a blue diamond for each event and a red dot for the Cas9 control. Mismatches between the gRNA and the off-target sequence are in red. The critical ‘seed’ region is underlined. ^a^ n = 10, ^b^ n = 5, ^c^ n = 4.

Soybean is a paleopolyploid [23] and thus most genes have a homoeolog. For functional genomic studies, it would be beneficial if the CRISPR system could be used to target a homoeologous gene-pair singly and at the same time. To test this, the soybean genes Glyma01g38150 and Glyma11g07220 (orthologs of the *A. thaliana DDM1* gene) were targeted. Three gRNAs were designed; one to target Glyma01g38150 (01gDDM1), one to target Glyma11g07220 (11gDDM1), and a third to target both (01g + 11gDDM1). Both single-targeting gRNAs resulted in average indel frequencies greater than 70% (Figure 2). For 01gDDM1, eight events had indel frequencies between 87-97%. Two events only had indel frequencies of 1-2%, but these were still higher than the Cas9 control (0.14%). All but one of the 11gDDM1 events had indel frequencies greater than 95% (Figure 2). The 01gDDM1 gRNA was specific for the intended chr1 target, but the 11gDDM1 gRNA led to a small but detectable level (2-13%) of off-target modifications at the chr1 sequence (Figure 3).

Genetic modifications at both DDM1 genes were detected in events containing the 01g + 11gDDM1 gRNA, but the average indel frequency was only 21% for chr1 and 8.9% for chr11 (Figure 2). Average indel frequencies greater than 97% were observed in events targeting a different homoeologous gene pair Glyma04g36150 and Glyma06g18790 (*A. thaliana MET1* orthologs), suggesting that the lower indel frequency of the 01g + 11gDDM1 vector is due to the gRNA itself and not a result of targeting multiple genes at once.

It is noteworthy that unique insertions of the *A. rhizogenes* root-inducing (Ri) plasmid [GenBank: AJ271050] were present in two 11gDDM1 events. The Ri insertions were identified in 4.8% of the reads from event 3 and 79.2% of the reads from event 4. Both insertions are from the left-border end of the Ri plasmid, approximately 1 kb apart from each other. Cloning and sequencing of event 4 showed a 252-bp insertion from the Ri plasmid (Additional file 3). These results are particularly interesting since it should be possible to increase the chances of obtaining targeted insertions, as has been shown with other nuclease systems [24-27].

### Targeting *MIR* genes

MicroRNAs (miRNAs) are small RNA molecules responsible for regulating a wide range of processes in plants [28]. MicroRNAs are encoded by *MIR* genes that are typically short (~500 bp), non-coding sequences. These features, coupled with the genetic redundancy of *MIR* families, may decrease the likelihood of isolating *MIR* mutants in mutagenesis screens [29]. Thus, the specific targeting of Cas9, and the large number of targets for any given gene, may make the Cas9 system well suited for generating *MIR* mutants. Two soybean miRNAs, miR1514 and miR1509 were targeted with Cas9. The short length of the *MIR* genes limited the number of possible Cas9 targets. Finding a *MIR1514* target near the mature miRNA was particularly difficult. Since mismatches are tolerated on the 5′ end of the gRNA [13], a C to G mismatch between the target and gRNA was made on the 5′ base (Figure 2) to get a target close to the mature miRNA. Indel frequencies greater than 95% were observed in all four miR1509-, and three out of four miR1514-targeted events. None of the short deletions (1-16 bp) were within the mature miRNA sequences, thus, none of the mutations are expected to alter the production of the miRNAs. However, these results demonstrate that short, non-coding sequences, such as *MIRs*, can be readily targeted by the CRISPR/Cas system.

### Genetic modification of somatic embryos

**Figure 4:**
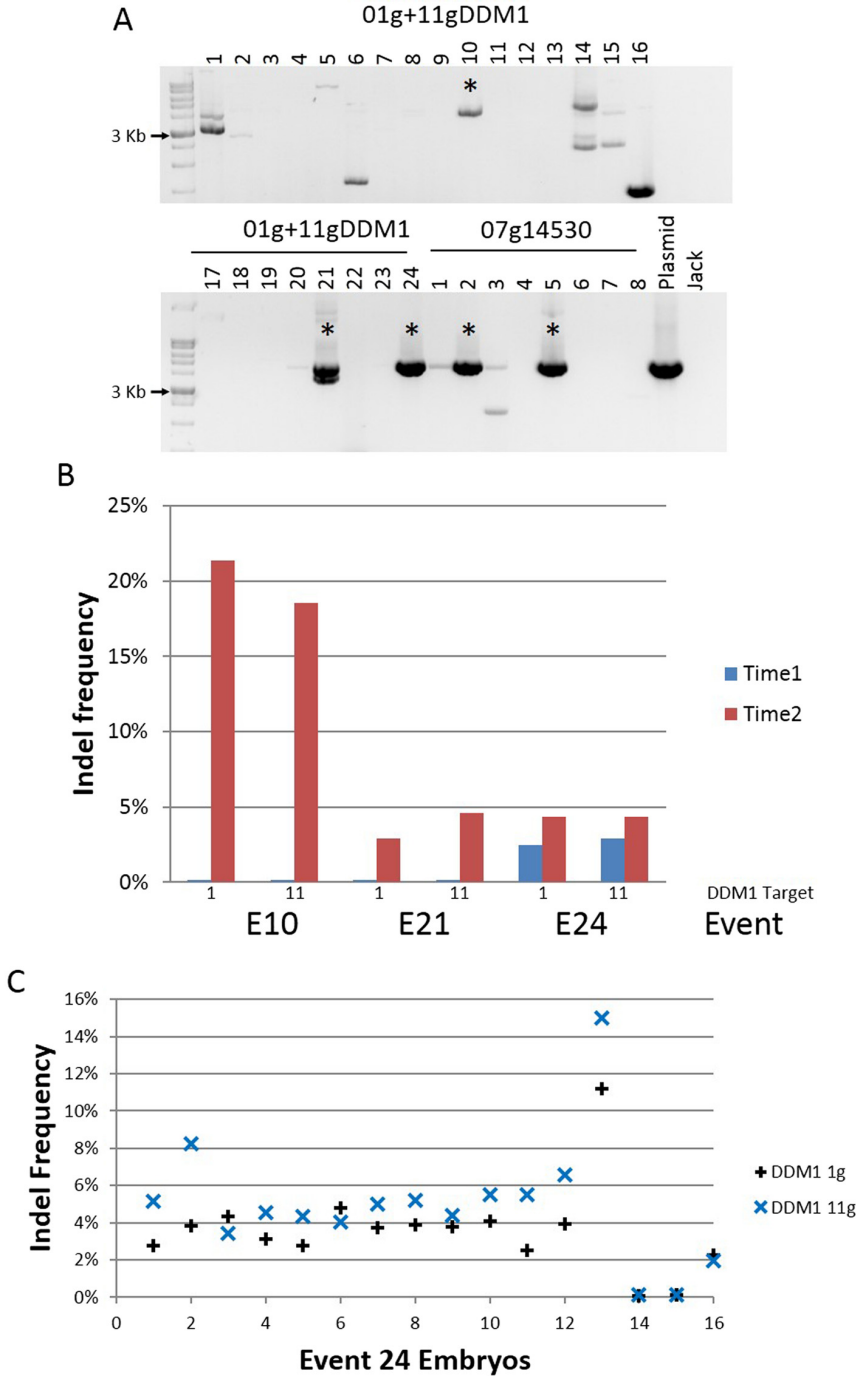
**DNA modifications in somatic embryos. (A)** Long-distance PCR for the Cas9 gene in recovered events with 01g + 11gDDM1 and 07g14530. Marker is a 1 Kb DNA ladder. Asterisks (*) indicate events with an intact Cas9. **(B)** Modifications were detected in three events transformed with the 01g + 11gDDM1 vector. At the initial time-point, modifications were only detected in event 24. When samples were taken approximately 2 weeks later, modifications were detected in all three events. **(C)** Modifications were detected in 14 out of 16 individual regenerating embryos from event 24.

Hairy roots are an excellent transgenic model system for soybean, however, they cannot generate whole plants, and therefore heritable mutations cannot be made. To evaluate CRISPR mutagenesis in whole plants, somatic embryo cultures of soybean were biolistically transformed with Cas9 constructs. Eight Glyma07g14530 and 24 01g + 11gDDM1 hygromycin-resistant events were recovered. Although each event contained portions of the gRNA and Cas9 genes as determined by PCR (data not shown), only two Glyma07g14530 and three 01g + 11gDDM1 events contained a complete Cas9 gene as determined by long-distance PCR (Figure 4A). When hairy-root events (*Agrobacterium* transformation) were screened, a full Cas9 product was observed in all ten events (Additional file 4A). These results suggest that the Cas9 gene fragmented during biolistic-mediated transformation, but not upon *Agrobacterium*-mediated transformation.

As with other Cas9 systems [10], the continued activity of Cas9 in the somatic embryos resulted in additional genetic modifications. DNA samples were taken from all events once there was enough tissue, approximately 2-4 weeks after selection, and used for amplicon sequencing. At this first sequencing time-point, event 24 had approximately 2.5 % modified sequences on chr1 and chr11, whereas events 10 and 21 had none. Although individual modified sequences made up fewer than 1% of the reads in event 24 (Additional file 2), such deletions were not observed in any of the other 23 events sequenced, indicating that these deletions were not due to sequencing errors. When DNA was collected approximately two weeks after the first sequencing experiment, the indel frequency increased to 4.3% in event 24. Events 10 and 21 had 20% and 4-5% modified sequences, respectively, for both targets (Figure 4B).

The two Glyma07g14530 events did not survive tissue culture and no modifications were detected in DNA from somatic embryos (data not shown). Individual embryos from event 24 range in indel frequency from 0-14%, with most of the events at 4% (Figure 4C). Therefore continued expression of Cas9 leads to additional mutations during the development of these embryos.

### Mutation efficiency

Of the nine targeting vectors used in this study, seven resulted in average indel frequencies greater than 70% (GFP 5′, 01gDDM1, 11gDDM1, Glyma04g36150, Glyma06g18790, miR1509, and miR1514). This mutation efficiency is ten-fold higher than the 3-7 % obtained with transcription-activator like effector nucleases (TALENs) in soybean hairy roots [30].

In hairy roots, the 01g + 11gDDM1 vector had the lowest average, with 21% and 8.9% for the chr1 and chr11 targets, respectively. A similar frequency was observed in the somatic embryos (Figure 4B, C). It should be noted that the 01g + 11gDDM1 gRNA is one base shorter than the rest of the gRNAs in this study (GN_19_GG). However, this target length has been used in plants [31], and shorter gRNAs (GN_18_GG) have been shown to be as effective as the commonly used gRNA (GN_20_GG) in cultured human cells [32]. It seems unlikely that a shorter gRNA led to a decrease it indel frequency, but a thorough testing of gRNA lengths in plants has not been reported. Although each of the vectors had a range of indel frequencies, only four out of 88 (5%) hairy-roots were unmodified, demonstrating that CRISPR mutagenesis in soybean is a robust system.

The three 01g + 11gDDM1 somatic-embryo events with the complete Cas9 gene contained targeted genetic modifications. These were three out of 24 hygromycin-resistant lines. These data demonstrate that when the complete Cas9 is incorporated, genetic modifications are made, although the complete Cas9 gene is only incorporated in 12.5% biolistically-transformed events. Of the recent reports of CRISPRs being used in plants, several have shown the recovery of whole-plants. One publication reported the biolistic transformation of rice, in which 9.4% and 7.1% of the T0 rice plants recovered contained mutations at their respective targets [31]. In this report, the Cas9 and gRNA cassettes were located on separate plasmids, and it is unclear if the complete Cas9 and gRNA cassettes were incorporated in all events. In contrast, transgenic *A. thaliana* and rice plants transformed with *Agrobacterium tumefaciens* had efficiencies of 20-90% for several targets [6,7,9,33]. Our data suggest that the disparity between biolistic and *Agrobacterium-*mediated transformation could be due to incomplete incorporation of the complete Cas9 gene upon biolistic-mediated transformation.

### Types of mutations

The types of mutations obtained here are similar to those observed in soybean and other plants obtained with ZFNs [15,21], TALENs [17,18,30] and CRISPRs [31,33-35]; small deletions were the most frequent mutations; SNPs were less common (Additional file 2).

The different targeting sequences tested led to a distinctive gamut of mutations. The seven most effective vectors almost exclusively generated short deletions, whereas the lower efficiency vectors contained more insertions/SNPs (Additional file 2). Of the ten 07g14530 events, seven had insertions of one or more bases. These results suggest that the differences were determined by either the target sequence or the gRNA. Therefore, multiple targeting vectors may be needed for any potential target sequence, depending on the frequencies/types of mutations desired. Obtaining a greater variety of mutations may be desirable when the intent is to produce an allelic series.

The types of mutations between the hairy-root events and somatic embryos are consistent between chromosomal targets and between transformation methods. Within the ten 01g + 11gDDM1 hairy-root events, six contained an A insertion on chr1 at the same position. From those same ten events, five contained an A insertion on the homoeologous target on chr11 (Additional file 2). Each of the somatic-embryo events has the same A insertion for both chr1 and chr11, and in many cases, it is the most abundant read (Additional file 2). Given the consistent insertion pattern, it is tempting to speculate that there may be rules governing the types of mutations that are possible for a given target.

### Evaluation of off-target modifications

One limitation of the CRISPR system is the potential for off-target modifications, i.e., the modification of sequences similar to the intended target sequence [13,36]. To determine the extent to which there may be off-target modifications, putative off-target sites were identified for the Glyma07g14530, DDM1, MET1, and miR1514 vectors. Each putative off-target site has two to six mismatches relative to the gRNA (Figure 3).

Two gRNAs created off-target mutations. The 11gDDM1 chr1 off target was modified in 2-13% of the sequenced reads, which is considerably lower than the indel frequency at the intended chr11 target (95-100%). When off-targeting occurred at miR1514 18g, there was a range of frequencies; 100%, 25%, and 5%. The 07g14530-15g and -17g off-target loci had indel frequencies of 2.8% and 2.2%, respectively. However, the increased indel frequencies were also observed in the Cas9 control, showing that they were due to sequencing errors caused by long stretches of T’s in the amplicons. These results indicate that while off-targeting does occur, at least for the tested gRNAs, it is not common, and was generally at a much lower frequency than at the intended target.

### gRNA vector construction

In this work, a rapid cloning method (Additional file 5) was developed to create new gRNAs. It consists of a single PCR reaction with two 41-bp primers and an In-Fusion® reaction and can be used to clone any gRNA target sequence. The pUC gRNA shuttle vector makes the construction of gRNAs simple and inexpensive. The use of the In-Fusion® cloning system has the benefit of reducing handling steps, to the point where it should be simple to automate the entire cloning process. Binary Cas9 vectors with four different selectable makers (*nptII*, GFP, hygromycin, *bar*) were also created to facilitate plant transformation experiments.

## Conclusions

This work shows that the Cas9 system is functional in two stably transformed plant systems, hairy roots and somatic embryos. It was possible to efficiently mutate all 11 loci chosen for testing; only two of the targeting vectors resulted in detectable off-target mutations at predicted off-target loci. The different gRNA targets produced different types of mutations. Combined with a vector system developed to efficiently assemble the necessary gRNAs, these results confirm that the CRISPR system will be a simple and inexpensive method for genome editing in soybean, thus facilitating the use of genome editing to confirm candidate genes, develop novel alleles/phenotypes, and engineer plants with important agronomic or quality traits.

## Methods

### Vector construction

The human codon-optimized Cas9 gene [2] was obtained from Addgene (plasmid 41815). Two flanking primers with added *Nhe*I and *Sac*II sites were used to amplify the coding sequence, including the SV40 nuclear localization signal, with the KAPA HiFi polymerase (KAPA BioSystems). The amplicon was digested with the two restriction enzymes and ligated to the vector, pM35S, between the double-enhancer 35S promoter and nopaline synthase (nos) terminator (Additional file 6). The entire cassette is flanked with I-*Sce*I restriction sites, which were used to move the Cas9 cassette into p201N to create p201N:Cas9 (Addgene plasmid 59175). The p201N vector is a p201BK [37] vector modified to include an *nptII* selectable marker cassette and I-*Sce*I and I-*Ppo*I restriction sites (Additional file 6).

For biolistic transformation of soybean, a pSMART HC Kan (Lucigen Corporation, [GenBank: AF532107]) cloning vector was modified to contain a *hygromycin phosphotransferase* (*hph*) gene under the control of the *Solanum tuberosum* Ubi3 promoter and terminator [38] and the meganuclease I*-Ppo*I site, and is referred to as pSPH2. The vector pSPH2 was digested with I-*Ppo*I and DNA overhangs were removed with T4 DNA polymerase. To prepare the Cas9 insert, p201N:Cas9:gRNA-Glyma07g14530 was digested with *Spe*I and *Pme*I and DNA overhangs were removed with T4 DNA polymerase. The vector and insert were ligated to create the plasmid pSPH2:Cas9:gRNA-Glyma07g14530. The Glyma07g14530 gRNA was then replaced with the 01g + 11gDDM1 (Glyma01g38150 and Glyma11g07220) gRNA via I-*Ppo*I to produce pSPH2:Cas9:gRNA-01g + 11gDDM1.

Additional binary Cas9 vectors were produced by replacing *npt*II from p201NCas9, with *hph*, *bar* (phosphinothricin resistance), or GFP. The *hph* cassette was moved from pSPH2 into the p201N Cas9 vector with the *Pac*I and *Spe*I restriction sites to produce p201H:Cas9 (Addgene plasmid 59176). The bar and GFP cassettes (double-enhancer 35S promoter, nos terminator) were amplified with the *Spe*I 35SF and *Pac*I nosR primers (Additional file 7), and moved into the p201N Cas9 vector with the *Pac*I and *Spe*I restriction sites to produce p201B:Cas9 (Addgene plasmid 59177) and p201G:Cas9 (Addgene plasmid 59178).

The gRNA targets were designed as previously described [2], with the exception of the U6 promoter, which was replaced with the *Medicago truncatula* U6.6 polymerase III promoter [39] for efficient transcription in soybean. For the gRNA targets, 22-23-bp targets were chosen that had the GN_19-20_GG motif as previously described [2]. The GFP 5′- and 3′-targets were chosen because they contain restriction sites that can be used for downstream analysis; however, given the high DNA-modification frequencies, such analyses were not performed. The GN_18-19_ portion of the genomic target motif was incorporated into the gRNA target molecule. The GFP, Glyma07g14530, and DDM1 gRNA target sequences were synthesized by IDT using gBlocks. The gBlocks were amplified by PCR with flanking primers containing I-*Ppo*I restriction sites. All primer sequences can be found in Additional file 7. The products were then digested with I-*Ppo*I and inserted into the p201N vector. The MET1 (Glyma04g36150 and Glyma06g18790), miR1514, and miR1509 gRNA target sequences were produced with the pUC gRNA shuttle vector system described below. Plasmids were electroporated into *Agrobacterium rhizogenes* strain K599 and used for hairy-root transformation. Vectors containing both the Cas9 and gRNA target cassettes were combined by inserting the gRNA target cassette into the p201N Cas9 I-*Ppo*I site.

### Hairy-root transformation of soybean

Soybean ‘Jack-GFP [40]’ and ‘Jack’ germinating seeds were used for transformation with slight modifications from the protocol previously described [41]. Briefly, soybean seeds were germinated for approximately one week under sterile conditions on a filter paper wetted with a ½ MSO liquid germination medium [42] supplemented with B5 vitamins [43]. *A. rhizogenes* (strain K599) containing the vectors-of-interest were streaked from glycerol stocks onto YM medium [44] supplemented with 50 mg L^−1^ kanamycin. Soybean cotyledons were prepared in a manner similar to that described for cotyledonary node transformation [45]; the root and lower hypocotyl were removed from the cotyledons, leaving approximately 5 mm of hypocotyl. The apical shoot and hypocotyl were cut longitudinally to produce two symmetrical cotyledons with a short hypocotyl piece. The apical meristem was removed and 1-mm-deep cuts were made in the cotyledons on the adaxial surface with a scalpel dipped in a solution of *A. rhizogenes* (PB Buffer (0.01 M Na2HPO4, 0.15M NaCl, pH 7.5) + 100 μM acetosyringone). Cotyledons were co-cultivated with *A. rhizogenes* for 3 days on filter paper wetted with 2 mL of liquid germination medium + 100 μM acetosyringone. Cotyledons were transferred to a hairy-root growth (HRG) medium according to Cho et al. [41] with the following modifications: ½ MS salts, 2 g L^−1^ Phytagel, and 500 mg L^−1^ timentin to inhibit *A. rhizogenes*. Each root was treated as an individual event and transferred to HRG medium with 10 mg L^−1^ of Geneticin (G418). Those roots that grew on HRG + G418 were considered events, and a 2-cm portion of a root tip was collected for CTAB DNA extraction [46]. PCR was performed to confirm the presence of the Cas9 and gRNA genes with the primers listed in Additional file 2. Long-distance PCR was performed with a Promega long-distance PCR master mix according to manufacturer’s instructions.

### GFP imaging

After selection on HRG + G418, root tips were imaged with an Olympus MVX10 microscope with a GFP filter cube and the imaging software DP controller version 2.2.1.227 (Olympus America Inc.). Blue-light images were taken with a 5 ms exposure.

### Custom-amplicon sequencing and analysis

Genomic DNA was amplified with the KAPA HiFi polymerase (KAPA Biosystems) with tailed primers under the conditions 95°C for 3 min; 30 cycles (98°C for 15 sec, 60°C for 15 sec, 72°C for 30-45 sec); and 72°C for 5 min. PCR products were run on a 1% agarose, 1X TBE gel and visualized on a UV transilluminator to verify amplification. PCR products were pooled across amplicons, diluted 1:100, and used as a template for a second PCR with the conditions 95°C for 3 min; 10 cycles (98°C for 15 sec, 60°C for 15 sec, 72°C for 30-45 sec); and 72°C for 10 min. The second PCR was used to add the final Illumina adapters and indexes. PCR products were again visualized to ensure amplification. All products were pooled and concentrated with DNA clean and concentrator columns (Zymo Research). The pooled samples were run on a 1.5% agarose, 1X TAE + cytidine gel and the proper fragments were gel extracted with the Zymoclean Gel DNA Recovery Kit. Purified libraries were quantified with the KAPA Library Quantification Kit (KAPA Biosystems) and run on an Illumina MiSeq (Illumina Inc.). Reads were de-multiplexed with the MiSeq reporter software version 2.3.32.

Reads were imported into the software Geneious (Biomatters Ltd.) version 7. Reads were trimmed for quality and separated by amplicon using the separate-reads-by-barcode function using the forward sequencing primer + five bases downstream as the barcode. The five downstream bases were essential to remove primer-dimers from the analysis. After quality and barcode trimming, only reads within five bases of the expected length were extracted for analysis. Reads were trimmed to regions approximately 20-bp upstream and downstream of the gRNA target site (Additional file 7). Sequences that were the length of wild-type sequences were extracted. Indel frequency was then calculated by subtracting the number of wild-type sequences from the total number of extracted reads.

For each of the targeted loci, unique sequences were extracted from the trimmed total extracted reads using the find-duplicates function. The most abundant, unique reads are reported in Additional file 2.

### Off-target sequence identification

Potential off-target sites were identified by comparing the 23-bp gRNA target sequences using BLAST to the soybean reference genome (Glyma v1.1), on Phytozome, setting the e-value threshold to 5 since the query sequence is only 23 nt. Only loci that had the required protospacer-adjacent motif (PAM) NGG motif at the 3′ end of the sequence were considered for analysis. Primers used for amplifying the off-target loci are in Additional file 7.

### Biolistic transformation of somatic embryos

Biolistic transformation of soybean was performed as previously described [47]. DNA was isolated from somatic embryo cultures for PCR and custom-amplicon sequencing.

### gRNA shuttle plasmid

To facilitate the construction of gRNA targets, a shuttle plasmid was created that makes construction quick and inexpensive. The *Medicago truncatula* U6.6 promoter was fused to the gRNA scaffold [2], and the entire gRNA is flanked by I-*Ppo*I restriction sites. To produce a novel gRNA target, forward and reverse primers were designed with tails that encode the new target sequence (Additional file 5). Fifteen bp of homology on the primer tails allowed for In-Fusion® cloning (Clonetech Laboratories Inc., Mountain View, CA). After transformation, the new gRNA target molecule was inserted between the promoter and gRNA scaffold. Sanger sequencing was performed with the commonly used M13-reverse primer to confirm the sequence of the gRNA. I-*Ppo*I was then used to move the functional gRNA target cassette into a vector of choice. The pUC gRNA Shuttle plasmid can be obtained from Addgene (plasmid 47024).

## Additional files

Additional file 1:
**GFP imaging of modified GFP events and controls.** Each panel is an independent event and blue-light images were overlaid onto white-light images of roots. Scale bar is shown as 5mm and all images are taken with the same magnification. Additional file 2:
**Unique sequences from all events in this study.** The most abundant reads for each event are reported. The number of reads, the respective percentages, and the type of modification (∆) is listed for each event. Wild-type sequences are in green, dashes are deletions, SNPs are orange, insertions are red, replacements are orange, an insertion of Ri plasmid is pink, and an inversion is in purple. Additional file 3:
**Cloned sequence from modified 11gDDM1 event containing a 252-bp insertion of the Ri plasmid.** Red is gRNA target, underline is insertion. Additional file 4:
**Long-distance PCR for the Cas9 gene in somatic embryos and hairy-root events.** (A) All hairy-root events are positive for Cas9. (B) Events positive for Cas9 from Figure 4A were re-run together to get appropriate sizing. Three 01g + 011gDDM1 and two 07g14530 biolistic-events have the correct 4.3kb band. Additional file 5:
**Cas9/gRNA targeting and cloning scheme to produce gRNAs.** GN_20_GG motifs are identified in a genomic region of interest. Tailed forward and reverse primers are designed to amplify the entire 3 kb gRNA Shuttle Plasmid. The primer tails contain sequences for the target (blue) and share 15 bp of homology (X’s) for the In-Fusion® protocol. PCR products can then undergo In-Fusion® cloning, resulting in the creation of the gRNA Target Plasmid. The gRNA cassette is in the middle of a multiple-cloning site for easy transfer to a final vector. This pUC gRNA Shuttle plasmid can be used for plant modifications, but the cloning scheme will work for any gRNA target. Additional file 6:
**Vectors used in this study.** The plasmid p201N Cas9 gRNA is the binary vector used for hairy-root transformations. The pSPH2 Cas9 gRNA vector was used for biolistic transformation. The pUC Shuttle vector was used to create additional gRNA targets. The targets were moved into the binary or biolistic vectors via the I-*Ppo*I restriction sites. Additional file 7:
**Primers used in this study. Underline denotes restriction site.** X’s are 15 bp of homology between primers required for In-Fusion® Cloning. Blue nucleotides are part of the gRNA target.

## Abbreviations

GFP: Green-fluorescent protein
CRISPR: Clustered, regularly interspaced, short palindromic repeats
Cas: CRISPR associated
gRNA: Guide RNA
nt: nucleotide
DSB: Double-strand break
NHEJ: Non-homologous end joining
SNP: Single nucleotide polymorphism
miRNAs: microRNAs
bp: Base pair
ZFNs: Zinc-finger nuclease
TALENs: Transcription activator-like effector nucleases
nos: nopaline synthase
hph: hygromycin phosphotransferase
PAM: Protospacer-adjacent motif
