# Supplementary figures and images for: Targeted genome modifications in soybean with CRISPR/Cas9
Source: BMC Biotechnol. 2015 Mar 12;15:16. doi: 10.1186/s12896-015-0131-2 (PMC4365529; doi:10.1186/s12896-015-0131-2)

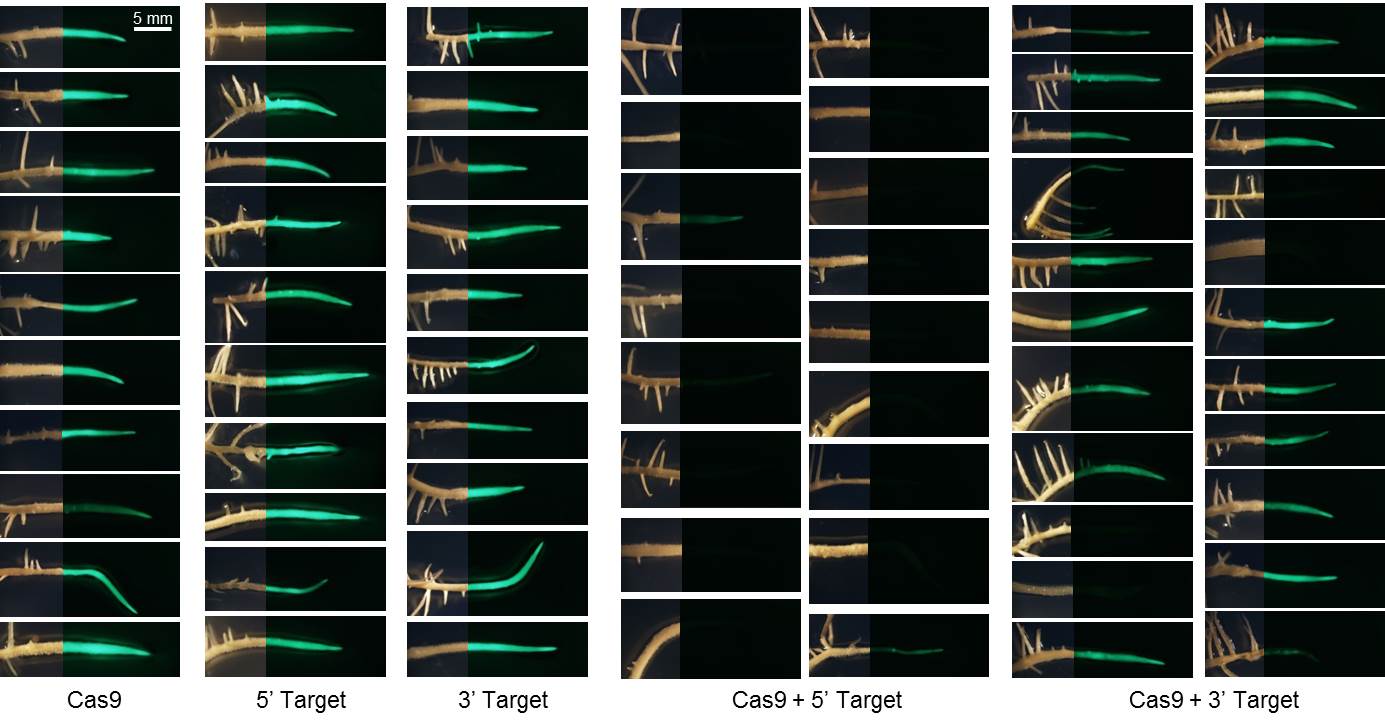

Supplement: Additional file 1: — GFP imaging of modified GFP events and controls. Each panel is an independent event and blue-light images were overlaid onto white-light images of roots. Scale bar is shown as 5mm and all images are taken with the same magnification. [file 12896_2015_131_MOESM1_ESM.jpeg]

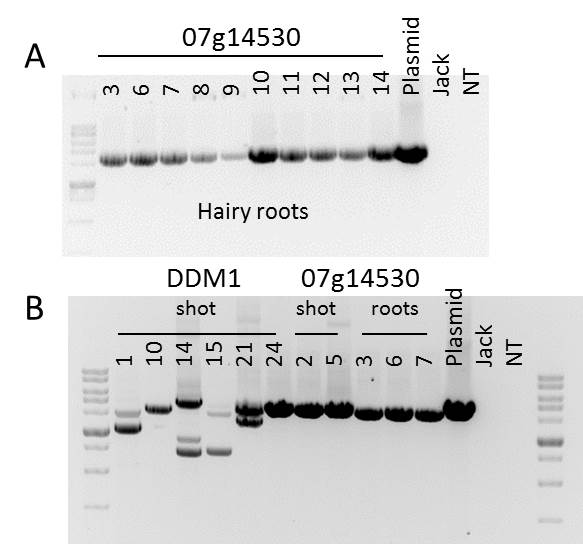

Supplement: Additional file 4: — Long-distance PCR for the Cas9 gene in somatic embryos and hairy-root events. (A) All hairy-root events are positive for Cas9. (B) Events positive for Cas9 from Figure 4A were re-run together to get appropriate sizing. Three 01g + 011gDDM1 and two 07g14530 biolistic-events have the correct 4.3kb band. [file 12896_2015_131_MOESM4_ESM.jpeg]

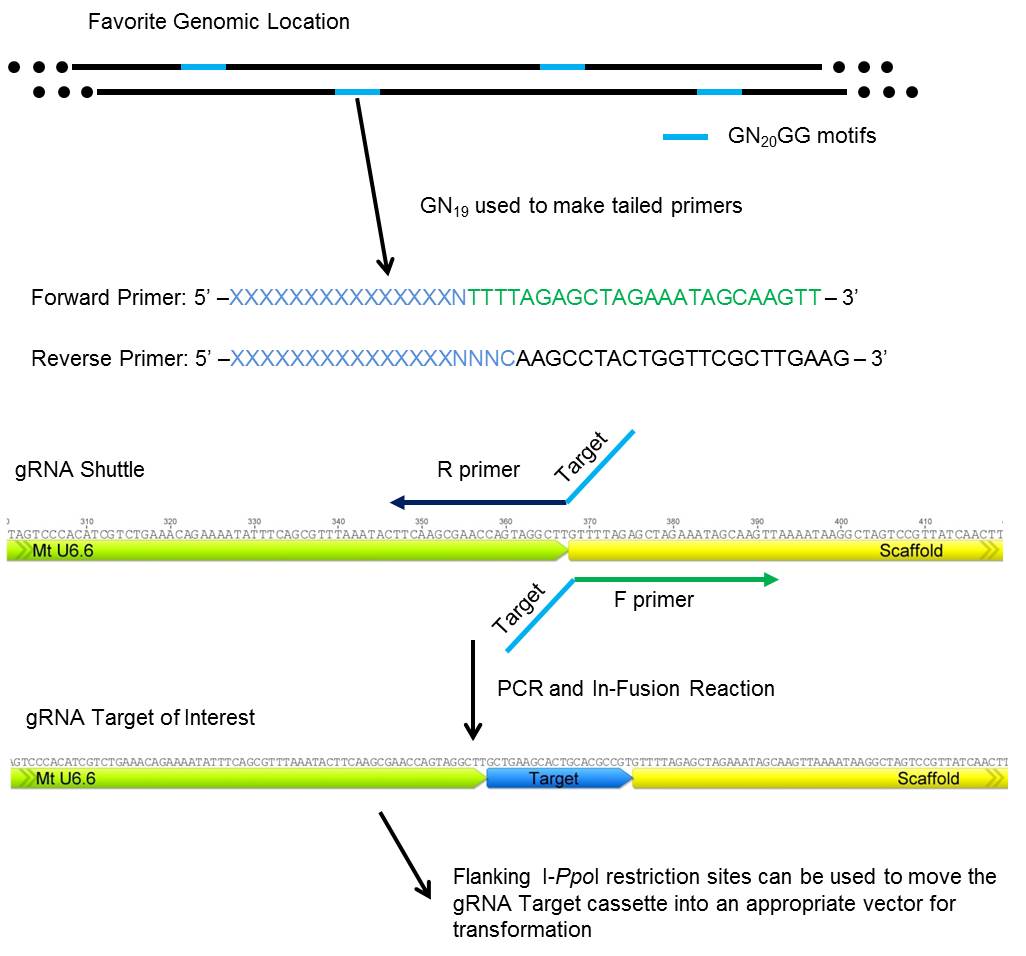

Supplement: Additional file 5: — Cas9/gRNA targeting and cloning scheme to produce gRNAs. GN20GG motifs are identified in a genomic region of interest. Tailed forward and reverse primers are designed to amplify the entire 3 kb gRNA Shuttle Plasmid. The primer tails contain sequences for the target (blue) and share 15 bp of homology (X’s) for the In-Fusion® protocol. PCR products can then undergo In-Fusion® cloning, resulting in the creation of the gRNA Target Plasmid. The gRNA cassette is in the middle of a multiple-cloning site for easy transfer to a final vector. This pUC gRNA Shuttle plasmid can be used for plant modifications, but the cloning scheme will work for any gRNA target. [file 12896_2015_131_MOESM5_ESM.jpeg]

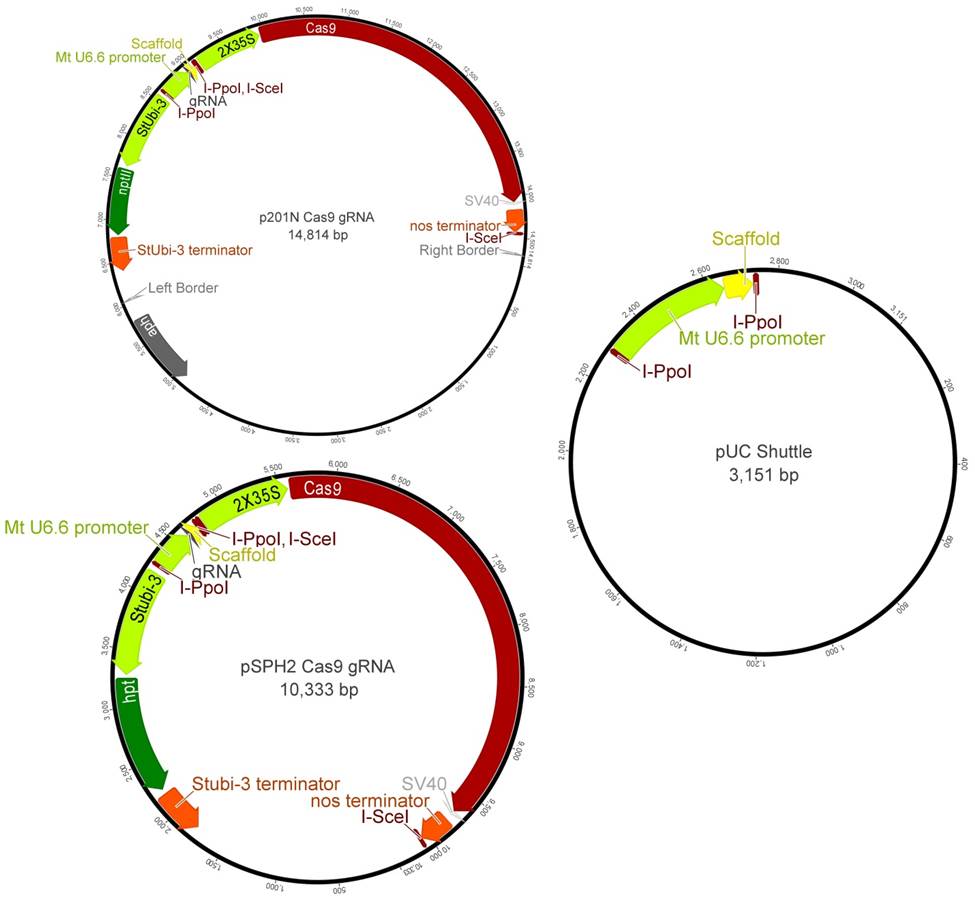

Supplement: Additional file 6: — Vectors used in this study. The plasmid p201N Cas9 gRNA is the binary vector used for hairy-root transformations. The pSPH2 Cas9 gRNA vector was used for biolistic transformation. The pUC Shuttle vector was used to create additional gRNA targets. The targets were moved into the binary or biolistic vectors via the I-PpoI restriction sites. [file 12896_2015_131_MOESM6_ESM.jpeg]
