# Supplementary material for: Targeted genome modifications in soybean with CRISPR/Cas9
Source: BMC Biotechnol. 2015 Mar 12;15:16. doi: 10.1186/s12896-015-0131-2 (PMC4365529; doi:10.1186/s12896-015-0131-2)
Supplement: Additional file 3: — Cloned sequence from modified 11gDDM1 event containing a 252-bp insertion of the Ri plasmid. Red is gRNA target, underline is insertion. [file 12896_2015_131_MOESM3_ESM.docx]

**NNNNNNNNNNTTNNNGTTCNATTTTTTCAATGNNNGGCTGTCTGTGCCCCAAAAGAAGAAGTTAAGTTGGAGGAAGAAGTGACTGCAGACATCAAAGATGATGGGACCTCTCTTATATCGAAAACAATGGTGGAGGAGGAAGAGAATTTAATTGAAGCTAGGATGAAGGAAGAGGAGGTACAACCAACGTCTTCGCCATACCGAATGAGGATGCTCTCTCTGCAATTCTTGGCGATCTCGGCGTGTTGTTGCCCGATCTGAGCAAGTGATCGCTCCGATCTGCCGCTGTTCTATTACATCCATAGTCACCGTAGGAAGGGAAAGAAGTCATAATTGCTTCAGTTGTTACTTAGTTTTTTCTATTTTAGTGTCTATGATCCGCCACTCCGGCGACTATTTCAATACACAACGCCAGAAATAATATAATATCTCCATGTGAGGAGGTACCTGACCTTAATGACACACAGTTTAACAAATTGGATGAGCTTTTGACTCAAACCAAACTGTACTCTGAGTTTCTGCTGGAGAAAATGGACGACATCACACTTGTAATTTTTAACCCTCTTTGCTTCTTTTTTTTTTCTGTGATAACTTTTTACACGTGTTGATTTGTTGTTCTTGTTGCATCATTCTTATTCTTTGAATATTTGTTGCTAGGCTGTGGGTGAACAAGAGAATAGGGAAAAGCAAGAGAGCAATCCTTCTGCAAAGAANAAGGGCTGTGGATCAAAAAGAA**
